# Distinct Role of TNFR1 and TNFR2 in Protective Immunity Against *Orientia tsutsugamushi* Infection in Mice

**DOI:** 10.3389/fimmu.2022.867924

**Published:** 2022-04-11

**Authors:** Yuejin Liang, James Fisher, Casey Gonzales, Brandon Trent, Galen Card, Jiaren Sun, Alexei V. Tumanov, Lynn Soong

**Affiliations:** ^1^ Department of Microbiology and Immunology, University of Texas Medical Branch, Galveston, TX, United States; ^2^ Institute for Human Infections and Immunity, University of Texas Medical Branch, Galveston, TX, United States; ^3^ Department of Pathology, University of Texas Medical Branch, Galveston, TX, United States; ^4^ Department of Microbiology, Immunology and Molecular Genetics, University of Texas Health Science Center at San Antonio, San Antonio, TX, United States

**Keywords:** Orientia tsutsugamushi, TNF, T cells, TNFR1, TNFR2, CD8

## Abstract

Infection with *Orientia tsutsugamushi*, an obligate intracellular bacterium, can cause mild or severe scrub typhus. Some patients develop acute lung injury, multi-organ failure, and fatal infection; however, little is known regarding key immune mediators that mediate infection control or disease pathogenesis. Using murine models of scrub typhus, we demonstrated in this study the requirement of TNF-TNFR signaling in protective immunity against this infection. Mice lacking both TNF receptors (TNFR1 and TNFR2) were highly susceptible to *O. tsutsugamushi* infection, displaying significantly increased tissue bacterial burdens and succumbing to infection by day 9, while most wild-type mice survived through day 20. This increased susceptibility correlated with poor activation of cellular immunity in inflamed tissues. Flow cytometry of lung- and spleen-derived cells revealed profound deficiencies in total numbers and activation status of NK cells, neutrophils, and macrophages, as well as CD4 and CD8 T cells. To define the role of individual receptors in *O. tsutsugamushi* infection, we used mice lacking either TNFR1 or TNFR2. While deficiency in either receptor alone was sufficient to increase host susceptibility to the infection, TNFR1 and TNFR2 played a distinct role in cellular responses. TNF signaling through TNFR1 promoted inflammatory responses and effector T cell expansion, while TNFR2 signaling was associated with anti-inflammatory action and tissue homeostasis. Moreover, TNFRs played an intrinsic role in CD8^+^ T cell activation, revealing an indispensable role of TNF in protective immunity against *O. tsutsugamushi* infection.

## Introduction

Scrub typhus is an acute, febrile, and often fatal disease, caused by *O. tsutsugamushi* bacteria, which transmit to humans *via* infected *Leptotrombidium* mites (1). Scrub typhus patients often present with an eschar at the mite bite site, along with flu-like symptoms including fever, headache, pneumonitis, and lymphadenopathy (2). Without appropriate treatment, the disease can progress to multiple organ failure and death. Approximately one million people are infected globally every year (3); no licensed vaccines are currently available for scrub typhus. Both clinical observations and animal model studies have demonstrated strong type 1-skewed immune responses, accompanied by severe inflammatory infiltration in multiple organs during *O. tsutsugamushi* infection (4–7). These robust immune activities may represent a double-edged sword in scrub typhus. While innate and adaptive immunity contribute to host protection against infection (8–10), overzealous immune responses are detrimental to the host, leading to harmful tissue damage, especially in endothelium (11). Therefore, the orchestration of delicate immune responses is required for host protection and tissue homeostasis.

TNF-α is a pleiotropic cytokine mainly produced by macrophages/monocytes, T cells, and neutrophils; it is critical for inflammatory and immune responses in infectious diseases (12, 13). TNF-α can signal through two trimeric receptors, TNFR1 (CD120a, p55/p60) and TNFR2 (CD120b, p75/p80), and is involved in diverse cellular processes (cell activation, apoptosis, necrosis, etc.). While cellular expression of TNFR1 is nearly universal, the expression of TNFR2 is limited primarily to immune cells (14, 15). TNFR1 is considered a “death receptor” and harbors a death domain in the cytoplasmic portion of the receptor that is capable of activating nuclear factor-kappa B-mediated proinflammatory transcription, cell survival, or death pathways (16). In contrast, TNFR2 does not contain a death domain, and activation of TNFR2 is associated with promotion of cell survival (16). In regards to scrub typhus, TNF-α was increased in *O. tsutsugamushi*-infected macrophages *in vitro* (17, 18), restricting bacterial replication (19). But, serum TNF-α levels in scrub typhus patients were positively correlated with disease severity (20, 21). These findings imply that TNF-α signaling may contribute to bacterial control, acute tissue injury, and immunopathogenesis in scrub typhus. However, the precise role of TNF signaling in *O. tsutsugamushi* infection remains undefined. By using different strains of TNFR-deficient mice and different regimens of anti-TNF-α treatment, we provide the first evidence that uncovers an essential and distinctive role of TNF signaling in immune responses against *O. tsutsugamushi* infection.

## Materials and Methods

### Mouse Infection and Ethics Statement

Female C57BL/6 mice (stock #000664), B6.129S-*Tnfrsf1a^tm1Imx^ Tnfrsf1b^tm1Imx^*/J mice (stock #003243), C57BL/6-*Tnfrsf1a* ^tm1Imx^/J (#003242), as well as B6.129S2-*Tnfrsf1b* ^tm1Mwm^/J (#002626) (22), were purchased from the Jackson Labs. Mice were maintained under specific pathogen-free conditions and used at 6-9 weeks of age, following protocols approved by the Institutional Animal Care and Use Committee (protocol # 1902006) at the University of Texas Medical Branch (UTMB) in Galveston, TX. All mouse infection studies were performed in the ABSL3 facility in the Galveston National Laboratory located at UTMB; all tissue processing and analysis procedures were performed in the BSL3 or BSL2 facilities. All procedures were approved by the Institutional Biosafety Committee, in accordance with Guidelines for Biosafety in Microbiological and Biomedical Laboratories. UTMB operates to comply with the USDA Animal Welfare Act (Public Law 89-544), the Health Research Extension Act of 1985 (Public Law 99-158), the Public Health Service Policy on Humane Care and Use of Laboratory Animals, and the NAS Guide for the Care and Use of Laboratory Animals (ISBN-13). UTMB is a registered Research Facility under the Animal Welfare Act and has a current assurance on file with the Office of Laboratory Animal Welfare, in compliance with the NIH Policy.

Mice were inoculated intravenously (*i.v.*) with 6 × 10^4^ FFU (200 µl) of *O. tsutsugamushi* Karp strain or PBS (mock) and monitored daily for weight loss, signs of disease, and survival (5). Some mice received anti-TNF-α antibody (200 μg/mouse, BioXcell) or IgG (200 μg/mouse, BioXcell) at 1, 3, 5, and 7 days post-infection, respectively. To neutralize TNF-α at a late stage, antibodies were administrated every day starting from D6 until the end of experiments. Serum and tissue samples (n = 4-5) were collected at D3 and D8, respectively, and inactivated for immediate or subsequent analyses. After *O. tsutsugamushi* infection, the following disease scores were used: Score 0: Normal behavior (typically seen with uninfected mice); Score 1: Active, <5% body weight loss, but eat and/or drink normally; Score 2: 6-10% weight loss, some ruffled fur (limited to areas between the shoulders), but eat and/or drink normally; Score 3: 11-19% weight loss, more pronounced ruffled fur, hunched posture, erythema, showing signs of reduced food/water taken; Score 4: 20-25% weight loss, decreased activity, bilateral conjunctivitis, showing signs of incapable to reaching food/water; Score 5: non-responsive or > 25% weight loss, a stage that the animal was humanely euthanized.

### Quantitative PCR for Measuring Bacterial Loads

To determine bacterial loads, portions of spleens and lungs were collected and homogenized by using a FastPrep-24 homogenizer (MP Biomedical). DNA was then extracted using a DNeasy Blood & Tissue Kit (Qiagen) and used for qPCR assays, as previously described (5). The primers were OtsuF630 (5’-AACTGATTTTATTCAAACTAATGCTGCT-3’) and OtsuR747 (5’-TATGCCTGAGTAAGATACGTGAATGGAATT-3’) (Integrated DNA Technologies). Bacterial loads were normalized to total nanogram (ng) of DNA per µL for the same sample, and data are expressed as the gene copy number of 47-kDa protein per ng of DNA. The copy number for the 47-kDa gene was determined by known concentrations of a control plasmid containing single-copy insert of the gene. Gene copy numbers were determined *via* serial dilution (10-fold) of the *O. tsutsugamushi* Karp 47-kDa plasmid.

### ELISA

Tissue proteins were extracted with a RIPA lysis buffer (Cell Signaling Technology) and quantified with a BCA Protein Assay kit (Thermo Fisher Scientific). Blood samples were collected in blood separation tubes (BD Bioscience) and centrifuged at 13,000 rpm for 2 mins to prepare serum samples. Protein and serum samples were inactivated *via* adding 0.09% sodium azide; TNF-α levels were measured *via* ELISA (Biolegend). For analysis of bacteria-specific T cell responses, splenocytes (5 × 10^5^/well) isolated from mice were seeded in 96-well plates with or without TSA56 protein (5 µg/mL). Supernatants were collected at 72 h and measured for IFN-γ production by ELISA (Biolegend).

### Flow Cytometry

Equivalent portions of lung tissues were harvested from mice, minced, and digested with 0.05% collagenase type IV (Thermo Fisher Scientific) in RPMI 1640 medium for 30 mins at 37°C. Minced tissues were loaded into Medicons and homogenized by using a BD Mediamachine System (BD Biosciences). Single-cell suspensions were made by passing cell homogenates through 70-µm cell strainers and treated with a Red Cell Lysis Buffer (Sigma-Aldrich). Leukocytes were stained with the Fixable Viability Dye (eFluor 506, Thermo Fisher Scientific) for live/dead cell staining, blocked with FcγR blocker, and stained with fluorochrome-labeled antibodies (Abs). For intracellular staining, cells were fixed and permeabilized using Foxp3/Transcription Factor Staining Buffer Set (Thermo Fisher Scientific). To evaluate antigen-specific T cell responses, lymphocytes were stimulated *ex-vivo* with TSA56 protein (10 µg/mL) overnight in the presence of brefeldin A for the last 4 h. The following Abs purchased from Thermo Fisher Scientific and BioLegend: PE-Cy7-anti-CD3ϵ (145-2C11), Pacific Blue-anti-CD4 (GK1.5), APC-efluor780-anti-CD8a (53-6.7), APC-anti-Ly6G (1A8-Ly6G), PE-Cy7-anti-CD80 (16-10A1), BV421-anti-CD206 (CO68C2), FITC-anti-CD64 (X54-5/7.1), Alexafluor700-anti-CD11b (M1/70), FITC-anti-CD69 (H1.2F3), BV711-anti-CD44 (IM7), APC-anti-CD62L (MEL-14), PE-anti-CD63 (NVG-2), Pacific Blue-anti-NK1.1 (PK136), PE-CF594-anti-NK1.1 (PK136). PE-anti-Foxp3 (FJK-16s), Percp-Cy5.5-anti-CTLA4 (UC10-4B9), APC-anti-TNFR1 (55R-286), and PE-anti-TNFR2 (TR75-89). Cells were fixed in 2% paraformaldehyde overnight at 4°C before analysis. Data were collected by a BD LSR Fortessa and analyzed *via* FlowJo software version 10 (BD Bioscience).

### Quantitative Reverse Transcriptase-PCR (qRT-PCR)

Tissues were homogenized using metal beads in a BeadBlaster 24 Microtube Homogenizer (Benchmark Scientific) with RLT lysis buffer (Qiagen). RNA was extracted using RNeasy Mini kits (Qiagen) and the synthesis of cDNA was proceeded using an iScript Reverse Transcription kit (Bio-Rad). cDNA was amplified in a 10 μL reaction mixture containing 5 μL of iTaq SYBR Green Supermix (Bio-Rad) and 5 μM each of gene-specific forward and reverse primers. The PCR assays were denatured for 30s at 95°C, followed by 40 cycles of 15s at 95°C, and 60s at 60°C, by utilizing the CFX96 Touch real-time PCR detection system (Bio- Rad). Relative quantitation of mRNA expression was calculated using the 2^−ΔΔCt^ method. The primers are listed in **Table S1**.

### Histopathology

All tissues were fixed in 10% neutral buffered formalin and embedded in paraffin. Tissue sections (5-μm thickness) were stained with hematoxylin and eosin. Sections were imaged under an Olympus BX53 microscope, and at least five random fields for each section were captured.

### Immunofluorescence

Lung tissues were fixed in 4% paraformaldehyde (EMS) for 24 h. Tissues were transferred into 20% sucrose/PBS for 24 h, followed by 30% sucrose/PBS for another 24 h. All fixations were performed at 4°C and tissues were finally frozen in O.C.T. compound (Sakura Finetek). Sections (8-μm) were processed in a humidified black box for fixing (with pre-chilled acetone for 10 min) and washing (with ddH_2_O for 5 times; TBS-0.025% Triton twice). After blocking, sections were incubated at 4°C overnight with rabbit anti-*O. tsutsugamushi* Karp serum (1:500) (6, 11), followed by the incubation with Alexa Fluor 555-conjugated anti-rabbit IgG (1:2,000, Life Technologies). All sections were stained with DAPI (1:5,000, Sigma-Aldrich). Sections stained with secondary Abs and DAPI only served as negative controls to optimize staining conditions. For each section, at least 6 fields of the lung sections were imaged on a Carl Zeiss Axio Observer fluorescence microscope (Carl Zeiss Microscopy LLC) equipped with ApoTome and Zen imaging software. Acquisition settings were identical among samples of different experimental groups and representative images are presented.

### Bioplex Assay

Cytokines and chemokine levels in mouse sera were measured by using a Bio-Plex Pro Mouse Cytokine 23-plex Assay kit (Bio-Rad) according to manufacturer protocol. Plates were washed using a Bio-Plex Pro II Wash Station and read using a Bioplex 200 system (Bio-Rad).

### Cell Purification and Adoptive Transfer

CD8^+^ T cells were purified from the spleens of naïve WT, TNFR1^-/-^, TNFR1^-/-^ and TNFR2^-/-^ mice by using MACS beads (Miltenyi Biotec) with the purity of 92-95%, based on flow cytometry (data not shown). At 1 day prior to bacterial infection, purified CD8^+^ T cells (2× 10^6^) were adoptively transferred into CD45.1 congenic recipient mice (n = 3) by *i.v.* injection. Mice were *i.v.* infected with bacteria the next day, and spleens were harvested at D8 for the analysis of T cell activation markers (CD44 and CD62L).

### Statistical Analysis

Data were presented as mean ± standard deviation (SD). Differences between individual treatment and control groups were determined by using unpaired Student’s t test, utilizing Welch’s correction when appropriate. One-way ANOVA was used for multiple group comparisons, with a Tukey’s *Post Hoc* for comparisons between groups. Statistically significant values are referred to as *, *p*<0.05; **, *p*<0.01; ***, *p*<0.001; and ****, *p*<0.0001, respectively.

## Results

### *O. tsutsugamushi* Infection in Mice Stimulated TNF-α and TNFR Expression

**Figure 1:**
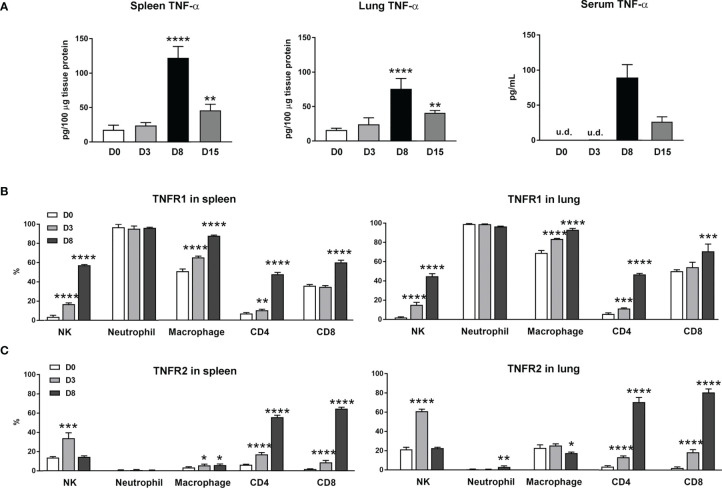
Increased expression of TNF-α and its receptors during *O. tsutsugamushi* infection. B6 mice (5/group) were injected *i.v.* with 6 × 10^4^ FFU of bacteria or PBS (used as a mock or D0). Spleen and lungs were collected for subsequent analysis at D3 and D8. **(A)** TNF-α protein levels in the spleen, lung and serum samples were analyzed by ELISA. **(B, C)** Surface TNFR1 and TNFR2 expression at D0, 3 and 8 on indicated immune cell subsets were analyzed by flow cytometry. Data are shown as mean ± SD from single experiments (n = 5) and are representative of two independent experiments. A one-way ANOVA with a Tukey’s multiple comparisons test was used for statistical analysis. *p<0.05; **p<0.01; ***p <0.001; ****p <0.0001.

Our previous studies of scrub typhus mouse models have demonstrated increased *Tnfa* mRNA levels in multiple organs at late stages of lethal infection (5, 11, 23). In this study, we used a comparable infection model and inoculation dose (**Figures S1A, B**). We found that TNF-α protein levels in the spleen, lung and sera peaked at day 8 (D8), with significantly high levels maintained at D15 when some animals were recovering (**Figure 1A**). Since immune functions of TNF-α are mediated *via* TNFR1 and TNFR2 (14), we profiled receptor expression on immune cell subsets by flow cytometry. We found diverse expression signatures of these two receptors among immune cells during infection, but near identical patterns between the spleen and lungs (**Figures 1B, C**). Uninfected mice had marginal TNFR1 or TNFR2 levels on T cell subsets. Infection significantly induced the percentages of receptor-expressing T cell subsets, with about 5- to 15-fold increases in TNFR2^+^ T cells in the spleen and lungs (**Figure 1C**). In contrast, TNFR1 but not TNFR2 was highly expressed on myeloid cells; infection further increased TNFR1^+^ macrophages (**Figure 1B**). Likewise, the proportions of TNFR1^+^ NK cells were significantly increased on D3 and D8 in both organs (**Figure 1B**), whereas TNFR2^+^ NK cells were increased only at D3 (**Figure 1C**). Therefore, *O. tsutsugamushi* infection promoted TNF-α protein production and induced differential expression profiles of TNFR1 and TNFR2 on innate versus adaptive immune cells, in a comparable manner between a lymphoid organ (spleen) and a major organ of infection (lung).

### TNFR1/2-Deficient Mice Were Highly Susceptible to *O. tsutsugamushi* Infection

**Figure 2:**
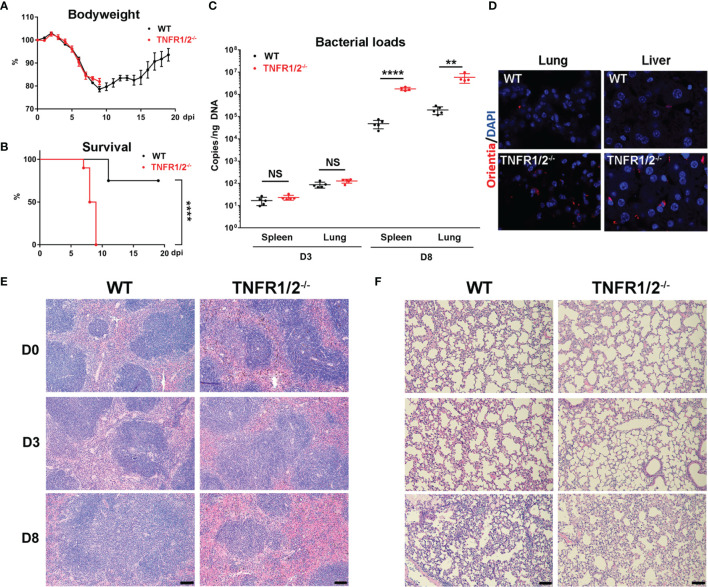
TNFR1/2-deficient mice were highly susceptible to *O. tsutsugamushi* infection. WT B6 and TNFR1/2-dificient mice were infected *i.v.* with 6 × 10^4^ FFU of bacteria. **(A)** Body weight and **(B)** survival curve (10 mice/group) were recorded daily. **(C)** Bacterial loads in the spleen and lungs at D3 and D8 were measured by qPCR (5 mice/group). **(D)** Immunostaining of bacteria (red color) in the lungs and liver. **(E, F)** H&E staining images of the spleen and lung were shown. Data are shown as mean ± SD from single experiments and are representative of two independent experiments performed. A two-tailed student t test was used for comparison of two groups. Survival data were analyzed using a log-rank (Mantel-Cox) test. **p<0.01; ****p <0.0001; NS, not significant.

To investigate the role of TNF-α signals in scrub typhus pathogenesis, we infected WT and TNFR1/2^-/-^ mice and monitored disease progression. Although body weight loss trends were comparable between two mouse groups, TNFR1/2^-/-^ mice were highly susceptible to infection, showing earlier death (D7 in knockouts vs. D11 in WT mice, **Figure 2A**), significantly higher lethality rates (100% at D9 in knockouts vs. 75% until D20 in WT mice, **Figure 2B**), and significantly higher bacterial loads in the spleen and lungs than WT mice at D8 (*p* < 0.01, **Figure 2C**). The immunostaining analyses confirmed bacterial qPCR data, showing readily detectable bacteria in TNFR1/2^-/-^ mouse tissues (**Figure 2D**). Infected TNFR1/2^-/-^ mice appeared to have smaller splenic white pulps (**Figure 2E**) and less pulmonary cellular infiltration at D8 (**Figure 2F**). These results demonstrate an essential role of TNFR signaling in host control of *O. tsutsugamushi* replication.

### Impaired Innate Cell Activation in Infected TNFR1/2-Deficient Mice

**Figure 3:**
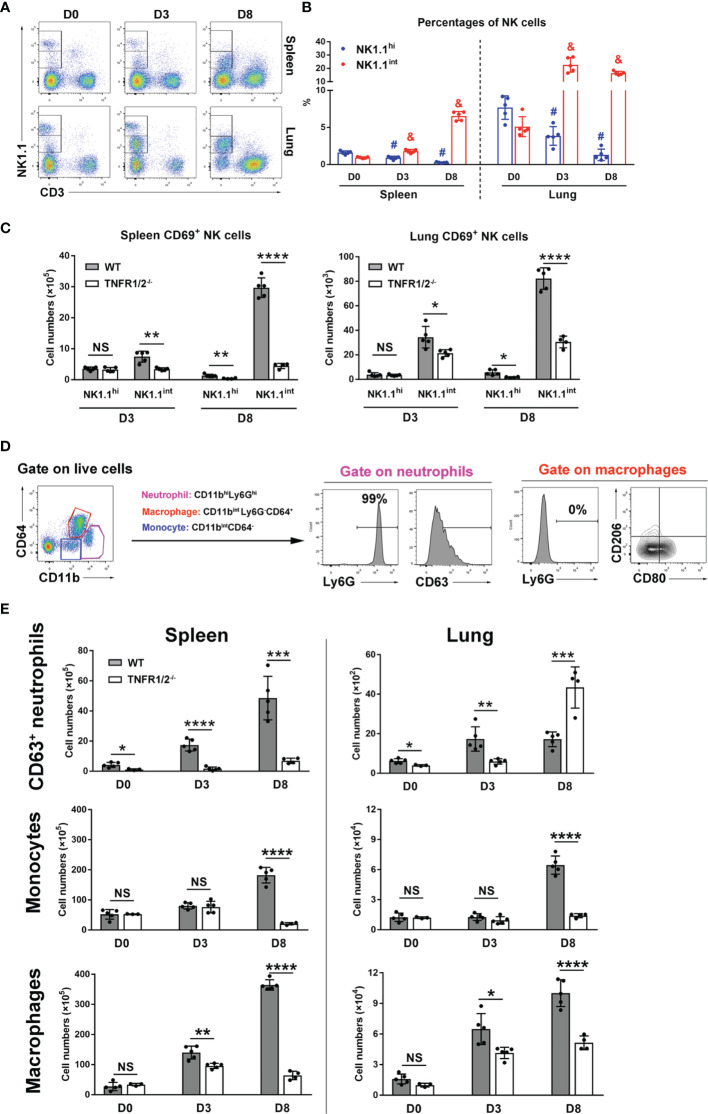
Deficiency of TNFR1/2 led to impaired innate immune responses. Mice (4-5/group) were infected and harvested, as in **Figure 2**. Leukocytes were isolated from the spleen and lung, followed by flow cytometry analysis. **(A, B)** NK cell subsets were gated on CD3^-^NK1.1^int^ and CD3^-^NK1.1^hi^. Dynamic pattern of NK cell percentages during infection were shown. **(C)** The numbers of CD69^+^ activated NK cells were measured in the spleen and lung. **(D)** Flow cytometry gating strategy of neutrophils, monocytes, and macrophages. CD11b^hi^Ly6G^hi^ subpopulation was identified as neutrophils and CD63 was a neutrophil activation marker. Almost all neutrophils were Ly6G^+^ cells. Macrophages were gated on CD11b^int^Ly6G^-^CD64^+^ cells. CD80 and CD206 were used for M1 and M2 markers respectively. Monocytes were characterized as CD11b^int^CD64^-^ cells. **(E)** The numbers of CD63^+^ neutrophils, monocytes and macrophages were shown. Data are shown as mean ± SD from single experiments and are representative of two independent experiments performed. A two-tailed student t test was used for comparison between two groups. ^#^ *p*<0.05 when compared to NK1.1^hi^ of D0; ^&^ *p*<0.05 when compared to NK1.1^int^ of D0; *p<0.05; **p<0.01; ****p<0.0001; NS, not significant.

NK cells are frontline cells in innate immunity, playing a critical role in infectious diseases *via* limiting pathogen spread and subsequent tissue damage (24). In scrub typhus patients, activated NK cells with increased IFN-γ production have been detected (25). Our flow cytometry experiments revealed two subpopulations of NK cells: NK1.1^hi^ and NK1.1^int^ cells (**Figure 3A**). While the NK1.1^hi^ subset predominated in uninfected mice and declined during infection, the NK1.1^int^ subset expanded significantly following infection (**Figure 3B**). Importantly, TNFR1/2^-/-^ mice not only had decreased numbers of activated CD69^+^ NK cells in the spleen and lungs at D8, but also decreased CD69^+^NK1.1^int^ cells at D3 (**Figure 3C**), indicating impaired NK cell maturation and activation in the absence of TNF receptors at early stages of infection.

Myeloid cells are target cells for *O. tsutsugamushi* infection and also act as defenders against bacteria (26). To understand how TNFR signals regulate myeloid cell functions, we analyzed neutrophils (CD11b^hi^Ly6G^hi^), macrophages (CD11b^int^Ly6G^-^CD64^+^), and monocytes (CD11b^int^CD64^-^) in WT and TNFR1/2^-/-^ mice by flow cytometry (**Figure 3D**). Almost all neutrophils expressed Ly6G, while macrophages were Ly6G negative (**Figure 3D**). As described in our previous studies (23, 26), CD63 and CD80 were used as activation markers for neutrophils and M1 macrophages, respectively, while CD206 was used as a M2 marker. As shown in **Figure 3E**, infection significantly increased and activated splenic neutrophils in WT mice. However, such responses were diminished or altered in TNFR1/2^-/-^ mice, as these mice displayed half the number of WT activated neutrophils in the lungs at D3, but double the number of infiltrated neutrophils at D8 (when animals developed obvious disease signs). Likewise, a significant decrease of monocytes and macrophages was found in both the spleen and lungs of knockout mice at D8 (**Figure 3E**). While *O. tsutsugamushi* infection resulted in predominate M1 macrophage polarization, as evidenced by high levels of CD80 expression, the percentages of CD206^+^ M2 macrophages were negligible (**Figure S2**). Downregulated CD80 expression on macrophages in TNFR1/2^-/-^ mice (**Figure S2**) suggested a vital role of TNF in regulating M1 macrophage activation. Thus, our findings suggest that TNF signaling is important for the expansion of myeloid cells in the lungs and spleen, mediating immune control of *O. tsutsugamushi.*

### T Cell Responses Were Severely Abrogated in the Absence of TNF Signaling During Infection

**Figure 4:**
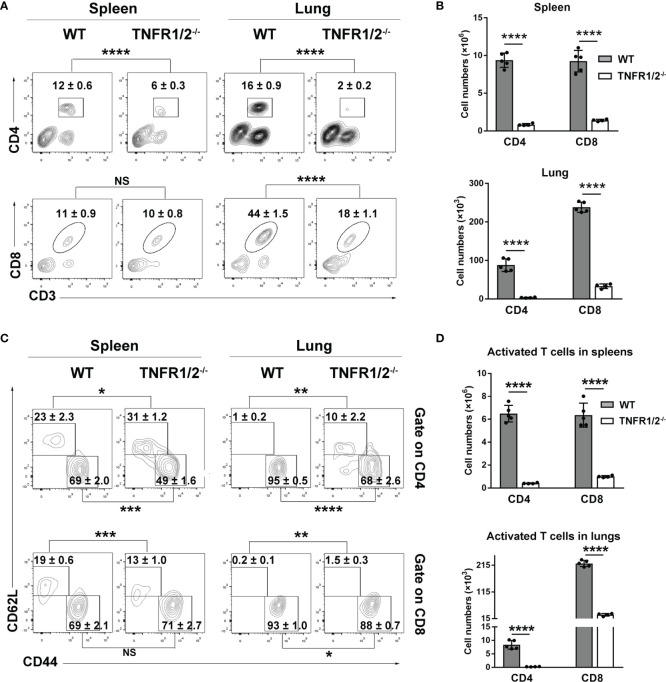
Deficiency of TNFR1/2 led to greatly impaired T cell responses in infection. Mice (4-5/group) were infected and harvested at D8, as in **Figure 2**. Leukocytes were isolated from spleens and lungs, stained, and analyzed by flow cytometry. **(A, B)** The percentages and numbers of CD4^+^ and CD8^+^ T cells. **(C, D)** The percentages and numbers of activated (CD44^+^CD62L^-^) CD4^+^ and CD8^+^ T cells. Data are shown as mean ± SD from single experiments and are representative of at least two experiments performed. A two-tailed student t test was used for comparison of two groups. *p<0.05; **p<0.01; ***p<0.001; ****p<0.0001; NS, not significant.

CD8^+^ T cells are essential for anti-*O. tsutsugamushi* immunity (9), but how TNF signaling contributes to the protective T cell immunity is unknown. To answer this question, we infected WT and TNFR1/2^-/-^ mice and analyzed T cell responses in the spleen and lungs. Although T cell activation (defined as CD44^+^CD62L^-^ phenotype) was comparable between two mouse groups at D3 (**Figure S3**), TNFR1/2^-/-^ mice displayed a significant and sharp decrease of T cell numbers at D8 compared to WT animals (**Figures 4A, B**). The CD4^+^ T cell activation was remarkably impaired in the absence of TNFR1/2, leading to very few CD4^+^ effector cells (**Figures 4C, D**). Similarly, the number of CD8^+^ effector T cells was also significantly lower in the knockouts, although the activation status was comparable between the two groups (**Figures 4C, D**). Of note, TNFR1/2^-/-^ mice displayed a 3-fold increase in the percentages of Foxp3^+^CD4^+^ T cells, as well as increased expression of CTLA4 (**Figure S4**), the latter is a key immune checkpoint molecule for suppressing effector T cell responses (27). These data suggest the central role of TNF signaling in T cell activation and population expansion during infection.

### Absence of TNFR1/2 Led to Higher Levels of Inflammatory Cytokines/Chemokines, but Decreased IFN-γ and IL-10 Production

**Figure 5:**
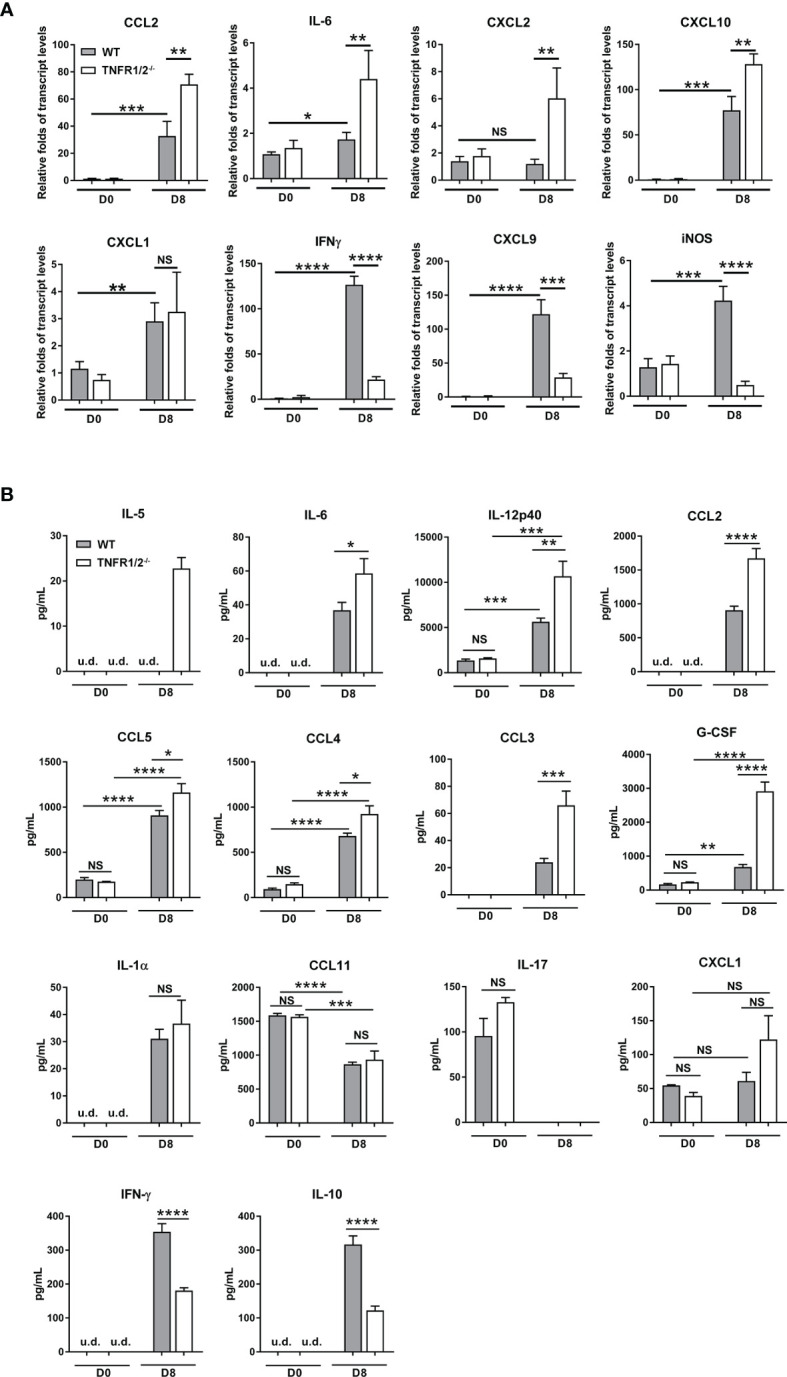
Deficiency of TNFR1/2 resulted in increased inflammatory cytokines/chemokines, but decreased IFN-γ and IL-10 in infection. Mice were infected, as in **Figure 2**. Tissues and blood were collected at D8 (4-5 mice/group). **(A)** qRT-PCR results of immune mediators in the lungs. **(B)** Bioplex assay of inflammatory cytokines in the sera. Data are shown as mean ± SD from single experiments and are representative of two independent experiments performed. A two-tailed student t test was used for comparison of two groups. *p<0.05; **p<0.01; ***p<0.001; ****p<0.0001; NS, not significant.

*O. tsutsugamushi* infection induces type 1-skewed inflammation in multiple organs, leading to immunopathogenesis and animal death (5, 23). To investigate whether TNF signaling modulates inflammatory responses, we measured cytokines/chemokines in the lungs and sera *via* qRT-PCR and Bioplex assays, respectively. TNFR1/2^-/-^ mice had increased transcript levels of *CCL2*, *IL-6*, *CXCL10*, and *CXCL2* (not *CXCL1*), but greatly decreased levels of *IFN-γ*, *CXCL9*, and *iNOS* in the lungs at D8 (**Figure 5A**). Elevated CCL2/3/4/5, IL-5, IL-6, IL-12p40 and G-CSF proteins were also detected in the sera of TNFR1/2^-/-^ mice, (**Figure 5B**). The infection also induced IL-1α production, but inhibited CCL11 and IL-17 in both WT and deficient mice. Consistent with the qRT-PCR result, TNFR1/2^-/-^ mice displayed a significant decrease in serum IFN-γ production, as well as a near 3-fold reduction of the immunosuppressive cytokine IL-10 (**Figure 5B**). Therefore, the lack of TNF receptors led to increased levels of inflammatory factors, but decreased production of IFN-γ and IL-10, suggesting a key role of TNF signaling in modulating tissue inflammation and orchestrating immune homeostasis.

### TNF-α Was Required for *O. tsutsugamushi*-Specific T Cell Responses and Bacterial Control

**Figure 6:**
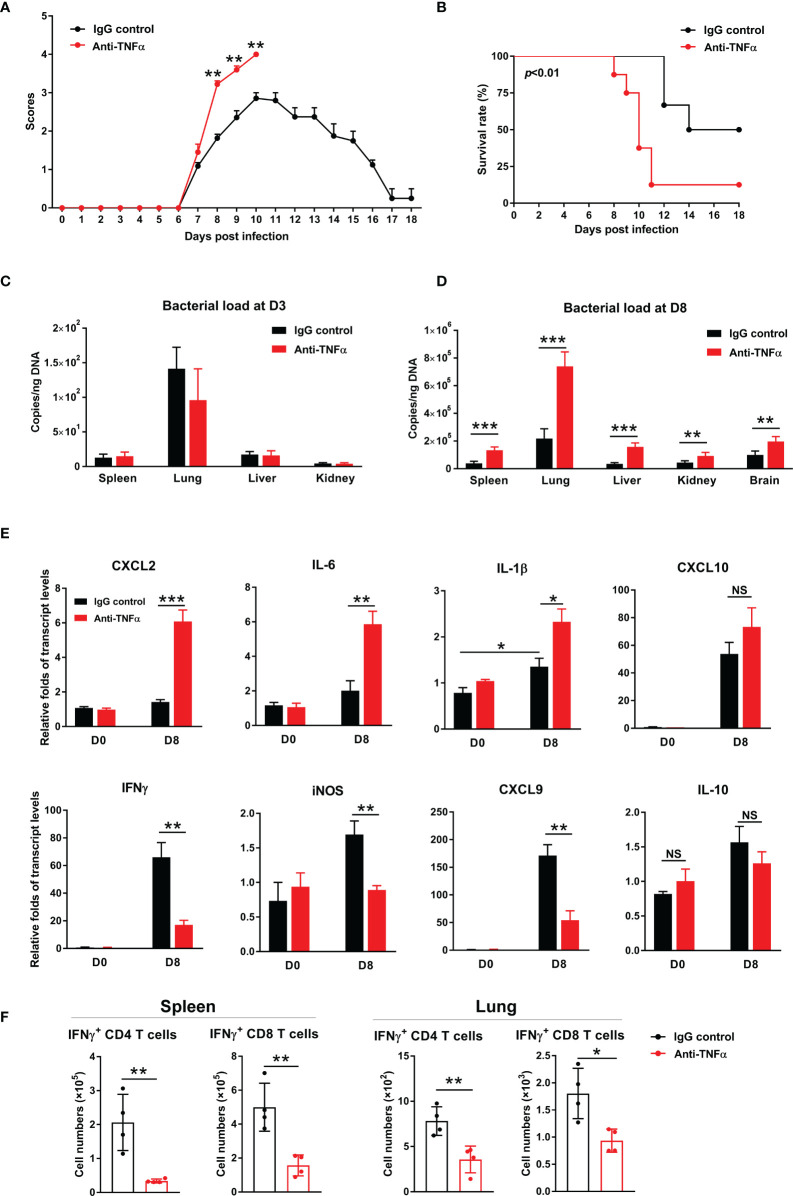
TNF-α neutralization increased host susceptibility to infection and exacerbated inflammatory responses. WT mice were infected *i.v.* with 6 × 10^4^ FFU of bacteria, as in **Figure 1**. Mice were treated with anti-TNF-α antibody every other day starting from D1 (200 µg/mouse, *i.p.*). Rat IgG was used as an isotype control. **(A)** Clinical scores and **(B)** survival curves (10 mice/group) were recorded daily. **(C, D)** Bacterial loads in indicated organs at D3 and D8 were measured by qPCR (4-5 mice/group). **(E)** Transcript levels of cytokines and chemokines at D8 were analyzed by qRT-PCR. **(F)** Lymphocytes were isolated from the spleen and lungs and stimulated with TSA56 protein (10 µg/mL) *ex vivo* overnight in the presence of brefeldin A for the last 4 h. Cells were harvested for intracellular staining of IFN-γ and analyzed by flow cytometry. Data are shown as mean ± SD from single experiments and are representative of two independent experiments performed. A two-tailed student t test was used for comparison of two groups. Survival data were analyzed using a log-rank (Mantel-Cox) test. *p<0.05; **p<0.01; ***p<0.001; ****p<0.0001; NS, not significant.

To confirm and extend above results, we treated bacteria-infected mice with an anti-TNF-α antibody at D1, 3, 5, and 7, respectively. We found that TNF-α neutralization significantly increased clinical scores from D8 to D10 (**Figures 6A, B**), as well as mortality (90% in treated mice vs 50% in isotype controls). Bacterial burdens were significantly increased in anti-TNF-α-treated mouse organs at D8, but not at D3 of infection (**Figures 6C, D**). Anti-TNF-α-treated mice had a 3-fold increase of *CXCL2* and *IL-6*, as well as one-fold increase in *IL-1β* in the lungs (**Figure 6E**). Consistent with TNFR1/2^-/-^ mouse results, neutralizing TNF-α caused a remarkable decrease of *IFN-γ*, *CXCL9*, and *iNOS* transcripts, while *IL-10* showed a decreased trend (**Figure 6E**). Furthermore, we evaluated antigen-specific T cell responses by intracellular cytokine staining. As shown in **Figure 6F**, TNF-α neutralization resulted in 3- to 4-fold decrease of antigen-specific IFN-γ-producing cells in the spleen, as IFN-γ-producing cell numbers were only half in the lungs of anti-TNF-α-treated mice compared with those of control animals. Therefore, our data demonstrated an essential role of TNF-α in *O. tsutsugamushi*-specific T cell immunity.

Although TNFR1/2 deficiency throughout infection or TNF-α blockage at early disease stages led to impaired immune responses and severe disease outcomes (**Figures 1**–**6**), it was unclear whether TNF is essential for pathogen control or for disease pathogenesis at late stages of infection (5). To address this important question, we modified our experimental strategy by treating bacteria-infected mice with TNF-α antibody daily from D6 (disease onset) to D10 (severe infection). We found that blockage of TNF signaling during this period did not alter body weight, clinical scores, and bacterial burdens as compared to control mice (**Figures S5A–C**). Comparable levels of inflammatory chemokines were also found between anti-TNF-α and control mice. These data demonstrated a dispensable role of TNF signaling in tissue inflammation at late stages of infection. In addition, *iNOS* transcript level was significantly downregulated in anti-TNF-α-treated mice, indicating that *iNOS*, which may contribute to bacterium killing (28), was highly dependent on TNF signals at both the early and late stages of infection (**Figures S5D** and **5A**) (29).

### Both TNFR1 and TNFR2 Contributed to Host Protection Against *O. tsutsugamushi* Infection

**Figure 7:**
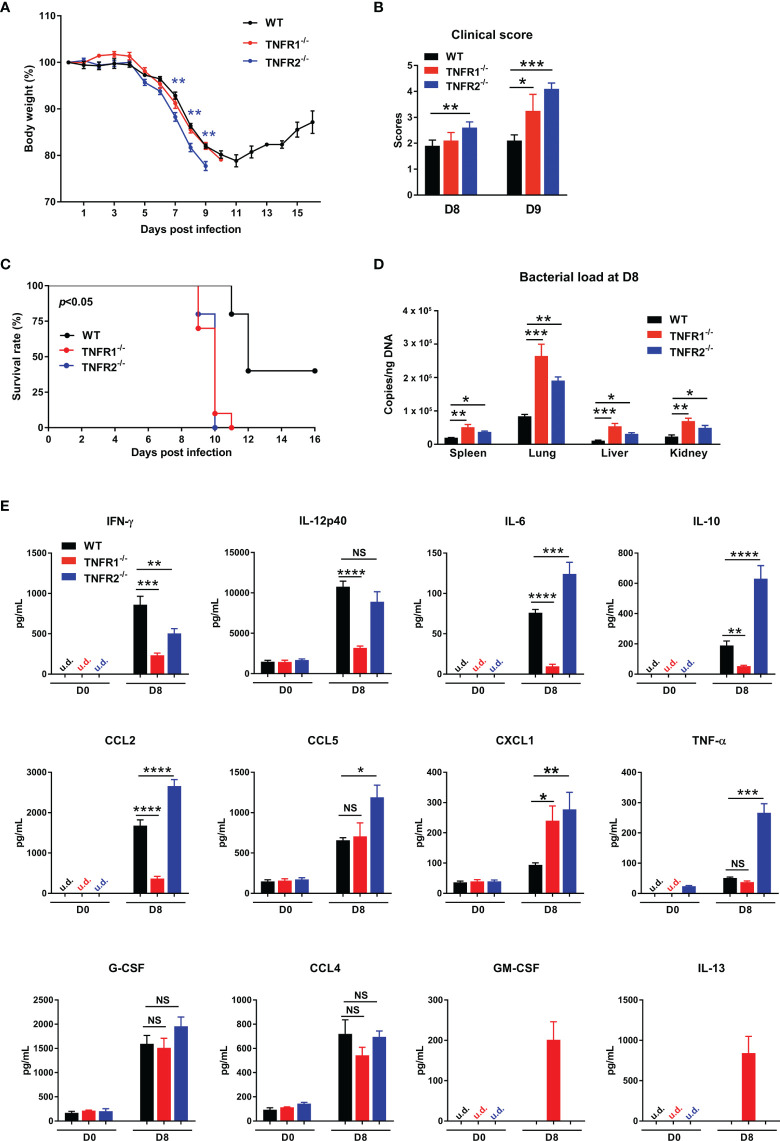
Both TNFR1 and TNFR2 were required for host protection against infection. WT, TNFR1^-/-^ and TNFR2^-/-^ mice (10/group) were infected *i.v.* with 6 × 10^4^ FFU of bacteria. **(A)** Body weight changes, **(B)** clinical scores, and **(C)** survival curves were recorded. **(D)** Bacterial loads in various organs at D8 were measured by qPCR (4-5 mice/group). **(E)** Serum chemokine/cytokine profile was analyzed by Bioplex assay. Data are shown as mean ± SD from single experiments and are representative of at least two experiments performed. A one-way ANOVA with a Tukey’s multiple comparisons test was used for statistical analysis. Survival data were analyzed using a log-rank (Mantel-Cox) test. Blue stars in **(A)** represent the comparison between WT and TNFR2^-/-^ groups. *p<0.05; **p<0.01; ***p<0.001; ****p<0.0001; NS, not significant.

TNFR1 and TNFR2 are involved in distinct signaling pathways and play diverse roles in immune regulation and cell function (14, 22). To understand how each TNFR contributes to host protection, we infected TNFR1^-/-^ and TNFR2^-/-^ mice and monitored daily. As shown in **Figure 7A**, weight loss started in all three groups of mice at D5; however, TNFR2^-/-^ mice developed more severe weight loss and disease scores than TNFR1^-/-^ or WT mice (**Figure 7A**). Notably, TNFR1^-/-^ mice were sicker than WT control, as evidenced by the higher clinical scores at D9, even though they had similar body weight loss (**Figure 7B**). Consequently, both TNFR1^-/-^ and TNFR2^-/-^ mice showed earlier mortality (D9) than WT animals (D11), and all succumbed prior to D11, when about 30% WT mice survived and began to recover (**Figure 7C**). We also analyzed tissue bacterial burden at D8 and found more than two- to three-fold increase of bacteria in all organs of TNFR2^-/-^ and TNFR1^-/-^ mice than WT mice (**Figure 7D**). Therefore, both TNFR1 and TNFR2 are required for bacterial control and host protection against *O. tsutsugamushi* infection.

To explore the underlying mechanisms, we measured cytokine/chemokine production in sera at D8. As shown in **Figure 7E**, both TNFR1^-/-^ and TNFR2^-/-^ mice produced less IFN-γ than WT controls, suggesting the requirement of both receptors for optimal type 1 immune responses. Additionally, CXCL1, a neutrophil migration chemokine, was upregulated in the absence of TNFR1 or TNFR2. Compared with WT mice, TNFR1^-/-^ mice had significantly lower production of IL-12p40, IL-6, IL-10, and CCL2, while TNFR2^-/-^ mice exhibited higher levels of IL-6, IL-10, CCL2, CCL5, and TNF-α. These distinct patterns of cytokine profiles indicated the proinflammatory role of TNFR1 and anti-inflammatory function of TNFR2, respectively. Of note, while GM-CSF and IL-13 were undetectable in infected WT mice, they were unexpectedly detected in TNFR1^-/-^, but not TNFR2^-/-^ mice (**Figure 7E**), suggesting a possible role of GM-CSF-mediated inflammation (30) or a dysregulation of type 2 responses in TNFR1^-/-^ mice (5, 31).

### Deficiency of TNFR1 or TNFR2 Resulted in Impaired Anti-Bacterial T Cell Responses

**Figure 8:**
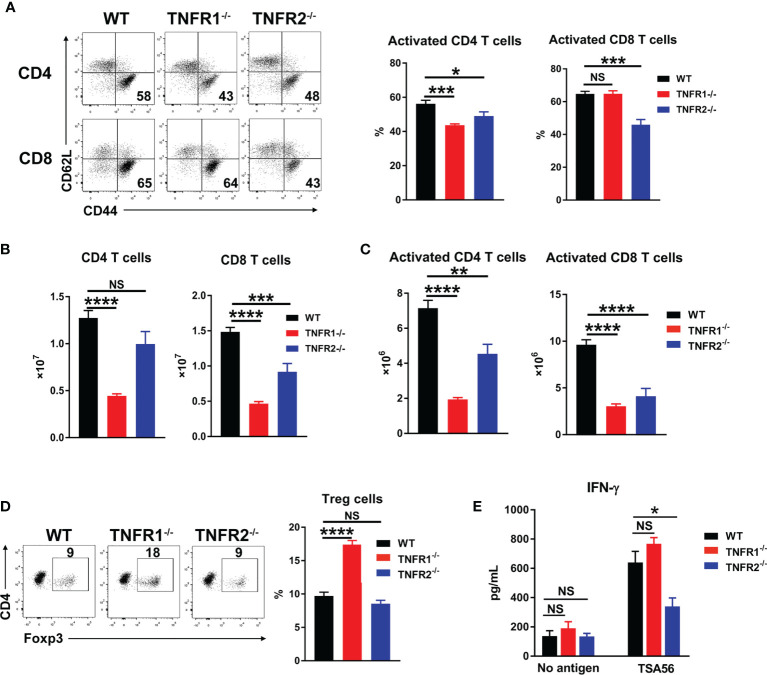
Lack of either TNFR1 or TNFR2 resulted in decreased anti-bacterial T cell responses. Mice (5/group) were infected, as in **Figure 7**. Lymphocytes were isolated from spleen and lungs at D8 and analyzed by flow cytometry. **(A)** The percentages of activated T cell subsets in the spleens. **(B)** The absolute numbers of T cell subsets in the spleens were counted and calculated according to flow cytometric analysis. **(D)** The percentages of regulatory T (Treg) cells. **(E)** Spleen cells (5 ×10^5^) were cultured in 96-well plate with or without 5 µg/mL of TSA56. Supernatants were collected at day 3, and IFN-γ production was measured by ELISA. Data are shown as mean ± SD from single experiments and are representative of at least two experiments performed. A one-way ANOVA with a Tukey’s multiple comparisons test was used for statistical analysis. *p<0.05; **p<0.01; ***p<0.001, ****p<0.0001; NS, not significant.

Adaptive T cell responses are crucial for *O. tsutsugamushi* control (8, 9). To examine whether TNFR1 or TNFR2 contributes to anti-bacterial T cell responses, we analyzed mouse splenic T cell activation at D8 by flow cytometry. As shown in **Figures 8A, B**, deficiency of either TNFR led to reduced CD4^+^ T cell activation as evidenced by decreased percentages of CD44^+^CD62L^-^ populations, while lack of TNFR2 also resulted in decreased CD8^+^ T cell activation. Notably, the absolute numbers of both pan T cells and effector T cells were significantly lower in TNFR1^-/-^ and TNFR2^-/-^ mice than those in WT mice (**Figures 8B, C**), indicating an important role of TNF signaling in promoting T cell responses (15). Interestingly, the absence of TNFR1, but not TNFR2, caused an increase of Foxp3^+^ regulatory T cells (**Figure 8D**), which may suppress T cell functions and inhibit anti-bacterial immune responses. To confirm this, we cultured splenocytes of infected mice with TSA56 protein for three days and measured IFN-γ levels in the supernatants. Comparable levels of IFN-γ were observed among three groups without TSA56 protein stimulation; however, immune cells from infected TNFR2^-/-^ but not TNFR1^-/-^ mice produced only half the amount of IFN-γ compared with those of WT mice (**Figure 8E**). Therefore, both TNFR1 and TNFR2 contributed to anti-bacterial T cell responses during infection, probably through different mechanisms: the lack of TNFR1 resulted in a sharp decrease of T cell numbers, whereas TNFR2-deficiency mainly led to reduced T cell activation accompanied with increased inflammatory cytokines.

### CD8^+^ T Cell Activation Was Dependent on TNFR1/2 Intrinsic Signals

**Figure 9:**
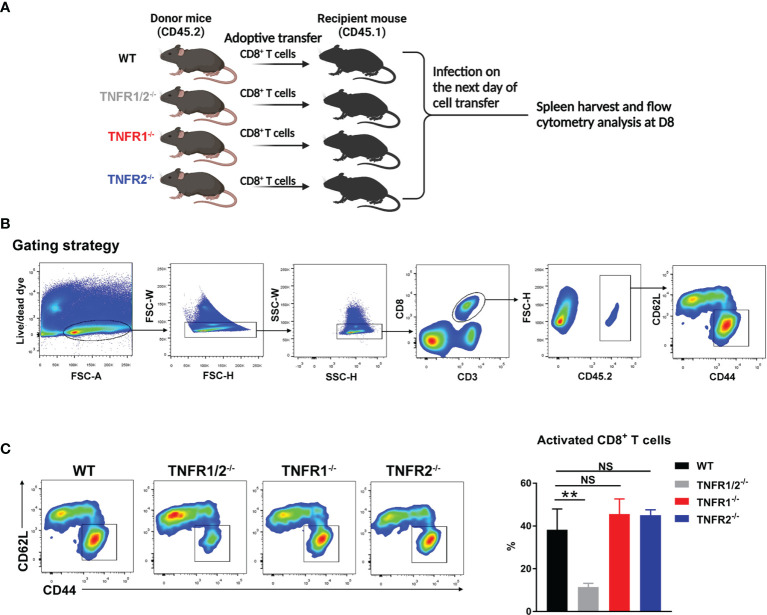
Intrinsic role of TNFRs in CD8 T cell activation. **(A)** CD8^+^ T cells were purified from the spleens of WT, TNFR1/2^-/-^, TNFR1^-/-^ and TNFR2^-/-^ mice by using MACS beads, followed by adoptively transferring into CD45.1 mice (2 ×10^6^ CD8^+^ T cells/mouse, 3 mice/group) at one day prior to infection. Mice were infected, as in **Figure 7**, and splenocytes were harvested at D8. **(B)** Flow cytometric gating strategy. Live and single spleen cells were gated first, followed by the gating of CD3^+^CD8^+^ T cells. The adoptively transferred CD8^+^ T cells from donor mice were identified by CD45.2^+^ populations. The donor cells were further analyzed for CD44 and CD62L, respectively. **(C)** The represented flow cytometry images and the percentages of CD8^+^ T cell activation (CD44^+^CD62L^-^) were presented. Data are shown as mean ± SD from single experiments and are representative of at two experiments performed. A one-way ANOVA with a Tukey’s multiple comparisons test was used for statistical analysis. **p<0.01; NS, not significant.

CD8^+^ T cells can protect the host against *O. tsutsugamushi* infection through cytokine production and/or cytotoxic effect (8, 9). Since TNFR1/2^-/-^ mice display defects in architecture of secondary lymphoid organs (22, 32), which may affect CD8^+^ T cell activation, we next tested whether intrinsic TNFR1/2 signaling contributes to CD8^+^ T cell activation. We purified naïve CD8^+^ T cells from CD45.2 donor animals (WT, TNFR1/2^-/-^, TNFR1^-/-^, or TNFR2^-/-^ mice) and adoptively transferred these cells into naïve CD45.1 recipients one day prior to *O. tsutsugamushi* infection (**Figure 9A**). Mice were euthanized on D8, and the donor cells in the spleens were first gated on CD45.2, followed by the evaluation of cell activation markers (**Figure 9B**). We found comparable percentages of activated CD44^+^CD62L^-^CD8^+^ T cells among WT, TNFR1^-/-^, and TNFR2^-/-^ groups, but a 3-fold reduction of activated CD8^+^ T cells in TNFR1/2^-/-^ mice (**Figure 9C**). Our results suggested that CD8^+^ T cell activation during *O. tsutsugamushi* infection was dependent on TNFR1/R2 intrinsic signals, while a deficiency in either TNFR1 or TNFR2 alone could be partially compensated by its partner receptor.

## Discussion

In addition to inducing inflammation and cell apoptosis, TNF-α is also involved in immune cell regulation critical for immunity to infectious agents (15). Clinical use of anti-TNF therapy in chronic inflammatory diseases can increase the risk of serious infections, implicating a key role of TNF in host immune defense (33). Animal infection studies for *Mycobacterium tuberculosis*, *Listeria monocytogenes*, and *Ehrlichia* have indicated that TNF-α is essential for pathogen control and host protection against intracellular bacteria (34–39). The serum TNF-α level in scrub typhus patients is considered a predictor of severe disease or death (20). In addition, *O. tsutsugamushi* infection suppressed macrophage-derived TNF-α (17, 40), which may inhibit bacterial growth *in vitro* (19). However, the role and underlying mechanism of TNF-α in host immune responses and immunopathogenesis in *O. tsutsugamushi* infection are poorly understood. By using neutralizing antibody *in vivo* and genetically modified mice, we provide strong evidence that TNF-TNFR signaling is required for boosting host immunity and controlling bacterial growth during scrub typhus. Our data demonstrated that the lack of TNF signaling at an early stage led to impaired innate cells (NK cells, macrophages, monocytes, and neutrophils) and adaptive immune responses (CD4 and CD8 T cells), accompanied by higher bacterial loads and increased mortality. We further revealed that both TNFR1 and TNFR2 contributed to host protection in *O. tsutsugamushi* infection, although they may achieve this through distinct mechanisms (**Figures 7**–**9**).

Consistent with observations in patient serum and our previous study in mice (5, 20), TNF-α protein levels peaked at D8 (**Figure 1A**), when the animals were sick. This elevation of TNF-α was also found in recovered mice at D15 (**Figure 1A**). TNF can signal through TNFR1 and TNFR2 to activate related downstream signaling pathways, leading to distinct target gene expression and regulation (14). We found that *O. tsutsugamushi* infection significantly increased TNFR1 and TNFR2 expression on T cells and NK cells, indicating that TNF might be important for lymphocyte activation and proliferation. In contrast, macrophages and neutrophils can constitutively express TNFR1, but not TNFR2 during infection (**Figures 1B, C**), implying that TNFR1 may selectively mediate myeloid cell activities.

To investigate the role of TNF signaling in *O. tsutsugamushi* infection, TNFR1/2^-/-^ mice were used in our study. These deficient mice were more susceptible to infection with higher bacterial loads in multiple organs at the peak of infection (**Figure 2**). Notably, neutralization of TNF-α at an early, but not late, stage disease reproduced the results from TNFR1/2^-/-^ mice, suggesting the importance of TNF signaling at the early stage of infection (**Figure 6**). Although bodyweight loss was comparable between these groups (**Figure 2A**), higher clinical scores were found in the absence of TNF (**Figure 6A**). Histopathological analysis showed that *O. tsutsugamushi* infection induced spleen dysregulation with enlargement of the splenic white pulp; however, deficiency of TNFR1/2 failed to elicit the same effect and caused smaller sizes of the spleen (data not shown), which suggests impaired immune cell expansion in lymphoid organs (**Figure 2E**). Thus, dampened immune cell expansion in lymphoid organs may cause poor immunity and uncontrolled bacterial growth in peripheral organs, leading to severe tissue damage and eventual animal death.

NK cells represent an important innate immune cell population in combatting several pathogens (24). Scrub typhus patients had increased NK cell numbers in peripheral blood, increased expression of the activation marker CD69, and enhanced IFN-γ secretion (25), implying a correlation between NK cell activation and disease severity (25). Our study here also revealed increased numbers of NK cells in WT mouse spleen and lungs during *O. tsutsugamushi* infection (**Figure 3**). Deficiency of TNFR1/2 resulted in greatly decreased numbers of CD69^+^ activated NK cells as early as D3, suggesting a key role of TNF signaling in regulating NK cell activation early during infection. One of our important findings was the discrimination of two NK cell subsets: NK1.1^hi^ and NK1.1^int^ cells (**Figure 3A**). These observations resemble the detection of CD56^hi^ and CD56^int^ NK cell subsets in the blood of scrub typhus patients (25). We found that NK1.1^int^ cell percentages were significantly increased and comprised the majority of NK cells with very few NK1.1^hi^ cells at D8 (**Figure 3**). These results correlate well with the reported increase of CD56^dim^ NK cells observed in scrub typhus patients (25). Given that different NK subsets in mice and humans may have distinct cellular cytotoxicity and cytokine production (41), further study is needed to elucidate the functional roles of these different cell populations in *O. tsutsugamushi* infection.

Myeloid cells can serve as target cells for *O. tsutsugamushi* replication, facilitating bacterial distribution from the site of infection to different organs and tissues, and as effector cells for eliminating bacteria by diverse defense machineries (23, 26). By using multi-color flow cytometry, we found decreased numbers of CD63^+^ activated neutrophils in the spleen and lungs of TNFR1/2^-/-^ mice at D3 (**Figure 3E**). Increased neutrophil numbers were also found in the lungs of TNFR1/2^-/-^ mice at D8, probably due to uncontrolled bacterial growth and relatively high levels of neutrophil chemokine CXCL2. Decreased numbers of macrophage/monocytes and reduction of CD80^+^ M1 macrophage polarization in deficient mice implied a critical role of TNF in macrophage survival, proliferation, and activation (**Figure S2**). Together, our data support a critical role for TNF in eliciting innate immune responses in *O. tsutsugamushi* infection.

Since TNFR1 and TNFR2 have distinct cellular distribution profiles and diverse biological functions (22), it is important to define the receptor-specific roles during *O. tsutsugamushi* infection (**Figures 7**–**9**). Surprisingly, deficiency in either TNFR1 or TNFR2 alone was sufficient to increase host susceptibility to infection (**Figure 7**). Like TNFR1/2^-/-^ mice, TNFR1 deficiency resulted in elevated bacterial burden, smaller spleen sizes, low IFN-γ and IL-10 levels (**Figure 7**), as well as very low levels of inflammatory cytokines (IL-6, IL-12p40, and CCL2) that were upregulated in TNFR1/2^-/-^ mice (**Figure 5**). Since TNFR2 was the only functional receptor for TNF-α signaling in TNFR1^-/-^ mice, this result suggested that TNFR2 might be preferably involved in immune modulatory functions during *O. tsutsugamushi* infection. Indeed, infected TNFR2^-/-^ mice produced higher levels of inflammatory mediators (IL-6, CCL2, CCL5, and TNF-α) and exhibited more severe disease outcome (increased weight loss and higher clinical scores) than their WT counterparts (**Figures 7A, B**). Interestingly, serum GM-CSF and IL-13 were only detected in TNFR1^-/-^ mice, but not in WT controls or TNFR2^-/-^ mice (**Figure 7E**). These data support a notion that TNFR2 signaling may evoke type 2-skewed immune responses (42), which are limited in murine models of scrub typhus (5, 43). The mechanisms underlying repressed type 2 responses during *O. tsutsugamushi* infection remain unknown and warrant further investigation.

It is known that CD44^+^ activated T cells may contribute to controlling bacterial growth through production of cytokines (e.g. IFN-γ and TNF-α) and granules (granzyme B) (9, 44), and that decreased CD4^+^ T cell numbers observed in acute scrub typhus patients is likely due to increased cell apoptosis (45). Our findings of reduced activated T cell numbers in TNFR1/2^-/-^ mice suggest a role of TNF-α signaling in promoting T cell activation following infection (**Figure 4**), since both TNFR1 and TNFR2 were essential for T cell activation and IFN-γ production *in vivo* (**Figures 7E**, **8**). The lack of TNFR1 alone caused impaired splenic T cell expansion that was also found in TNFR1/2^-/-^ mice, indicating that TNFR1 might be important for immune cell priming and subsequent proliferation. In contrast, TNFR2 was more important for T cell activation, evidenced by decreased activation *in vivo* and suboptimal IFN-γ secretion by *O. tsutsugamushi* protein stimulation *ex-vivo* (**Figures 7A, C**). Our findings of increased percentages of Foxp3^+^ regulatory T cells and CTLA4 expression (**Figure S4**) in TNFR1/2^-/-^ mice were consistent with previous reports, in which TNF-α promotes type 1 immunity through inhibiting regulatory T cell differentiation (46–48). A 2-fold increase of Treg cell percentages in TNFR1^-/-^ mice (**Figure 8D**) was also consistent with reports from other models, which have shown that excessive TNF signals through TNFR2 enhances Treg cell differentiation and function in inflamed tissues during infection (49–51). For efficient control of cytoplasmic bacteria like *O. tsutsugamushi*, CD8^+^ T cells are essential (8, 9). We first demonstrated the impaired CD8^+^ T cell activation in mice lacking TNF signals (**Figures 4** and **6**), and further proved that CD8^+^ T cells lacking TNFR1/2 were poorly activated compared with WT ones (10% v.s. 40%, *p* < 0.01), suggesting an intrinsic role of TNFRs in CD8^+^ T cell activation. However, TNFR1^-/-^ or TNFR2^-/-^ CD8 T cells were activated to the same magnitude of WT controls, indicating a possible compensation by counterpart receptor-mediated signaling during CD8^+^ T cell activation (**Figure 9**). Further study is warranted to elucidate the molecular mechanisms underlying TNF-α-mediated cytotoxic T lymphocyte responses, including cell survival, proliferation, cytokine production, and immune cell exhaustion during intracellular bacterial infection.

Together, this is the first detailed study demonstrating the requirement of TNF signals for both innate and adaptive immune responses against *O. tsutsugamushi* infection. Deficiency of TNF receptors, jointly or alone, significantly impaired the activation of innate and adaptive immunity, leading to uncontrolled bacterial growth and dissemination and significant increase of host susceptibility to this bacterial species. More importantly, our data reveal a distinct role of TNFR1 and TNFR2 in host immunity against *O. tsutsugamushi* infection; TNFR1 preferentially promotes immune cell activation and expansion, whereas TNFR2 plays an immunomodulatory role in inflammatory responses and tissue homeostasis. New therapeutic strategies targeting TNFR2 may help limit tissue inflammation and orchestrate T cell activities (52, 53) against *O. tsutsugamushi* infection.

## Data Availability Statement

The raw data supporting the conclusions of this article will be made available by the authors, without undue reservation.

## Ethics Statement

Mice were maintained under specific pathogen-free conditions and used at 6-9 weeks of age, following protocols approved by the Institutional Animal Care and Use Committee (protocol # 1902006) at the University of Texas Medical Branch (UTMB) in Galveston, TX. All mouse infection studies were performed in the ABSL3 facility in the Galveston National Laboratory located at UTMB; all tissue processing and analysis procedures were performed in the BSL3 or BSL2 facilities. All procedures were approved by the Institutional Biosafety Committee, in accordance with Guidelines for Biosafety in Microbiological and Biomedical Laboratories. UTMB operates to comply with the USDA Animal Welfare Act (Public Law 89-544), the Health Research Extension Act of 1985 (Public Law 99-158), the Public Health Service Policy on Humane Care and Use of Laboratory Animals, and the NAS Guide for the Care and Use of Laboratory Animals (ISBN-13). UTMB is a registered Research Facility under the Animal Welfare Act and has a current assurance on file with the Office of Laboratory Animal Welfare, in compliance with the NIH Policy.

## Author Contributions

YL and LS contributed to conception and design of the study. YL, BT, GC, and LS performed experiments, data collection and statistical analysis. YL and LS wrote the first draft of the manuscript. YL, JF, CG, LS, JS, and AT revised the manuscript. All authors contributed to manuscript revision, read, and approved the submitted version.

## Conflict of Interest

The authors declare that the research was conducted in the absence of any commercial or financial relationships that could be construed as a potential conflict of interest.

## Publisher’s Note

All claims expressed in this article are solely those of the authors and do not necessarily represent those of their affiliated organizations, or those of the publisher, the editors and the reviewers. Any product that may be evaluated in this article, or claim that may be made by its manufacturer, is not guaranteed or endorsed by the publisher.
