# Supplementary material for: Distinct Role of TNFR1 and TNFR2 in Protective Immunity Against Orientia tsutsugamushi Infection in Mice
Source: Front Immunol. 2022 Apr 11;13:867924. doi: 10.3389/fimmu.2022.867924 (PMC9035742; doi:10.3389/fimmu.2022.867924)
Supplement: Supplementary file 1 [file DataSheet_1.docx]

**
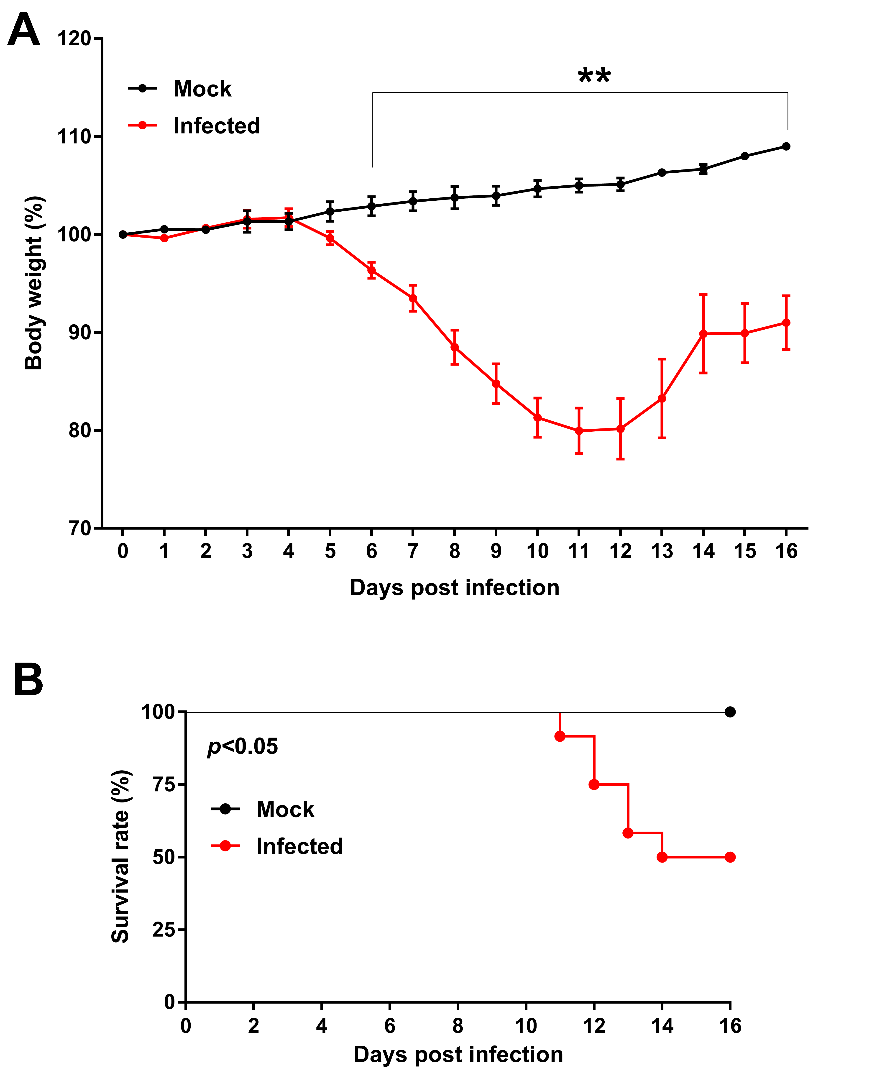
**

**Figure S1. The mouse model of *O. tsutsugamushi* infection**. B6 mice were *i.v.* injected either with *O. tsutsugamushi* (6 × 10^4^ FFU) or PBS (mock). The percentages of body weight loss (A) and survival rates (B) were recorded daily. A two-tailed student t test was used for comparison of body weight between the two groups. Survival data were analyzed by using a log-rank (Mantel-Cox) test. **, *p*<0.01.

**
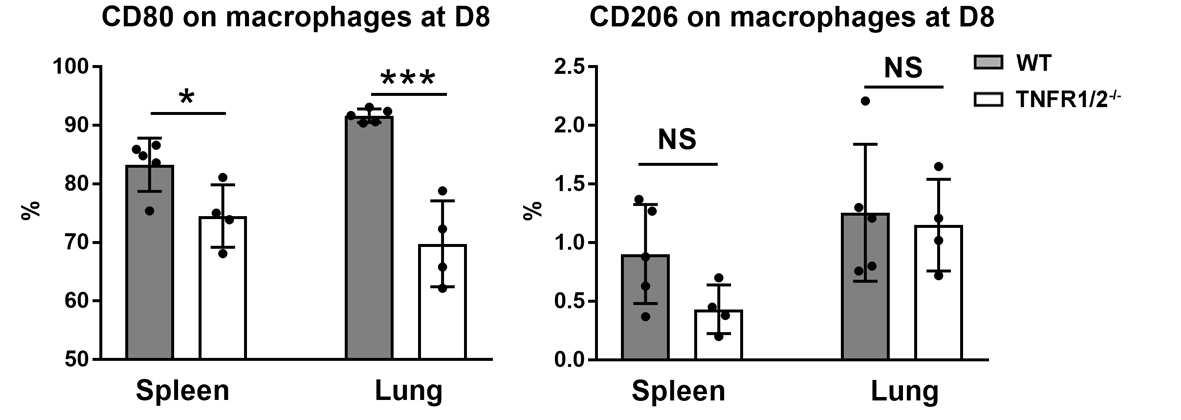
**

**Figure S2. Decreased M1 macrophages in the lungs of TNFR1/2-deficient mice.** WT and TNFR1/2^-/-^ mice were infected *i.v.* (6 × 10^4^ FFU of bacteria) and sacrificed at D8 (4-5 mice/group). Spleen- and lung-derived leukocytes were stained and analyzed by flow cytometry. The percentages of M1-like (CD80^+^) and M2-like (CD206^+^) macrophages were measured. Data are presented as mean ± SD from single experiments. Shown are representative data from at least two independent experiments. A two-tailed student t test was used for comparison of two groups. *, *p*<0.05; ***, *p*<0.001; NS, not significant.


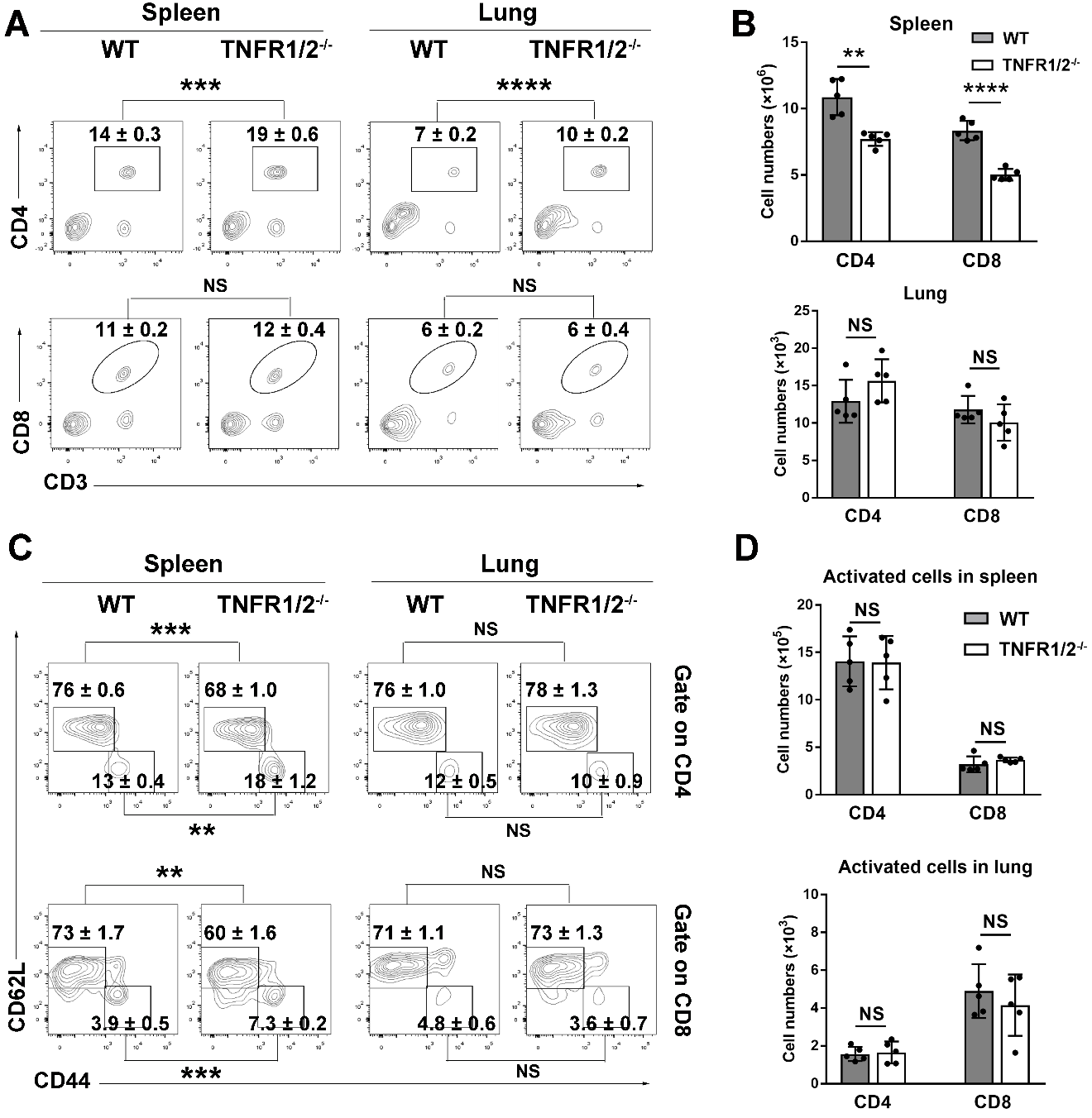


**Figure S3. Comparable T cell responses in WT and TNFR1/2-deficient mice at D3.** WT and TNFR1/2-deficient mice were infected *i.v.* with bacteria (6 × 10^4^ FFU) and sacrificed at D3. Spleen- and lung-derived lymphocytes were stained and analyzed by flow cytometry. (A-B) The percentages and total numbers of CD4^+^ and CD8^+^ T cells. (C-D) The percentages and total numbers of activated (CD44^+^CD62L^-^) CD4^+^ and CD8^+^ T cells. A two-tailed student t test was used for comparison of two groups. **, *p*<0.01; ****, *p*<0.0001; NS, not significant.

**
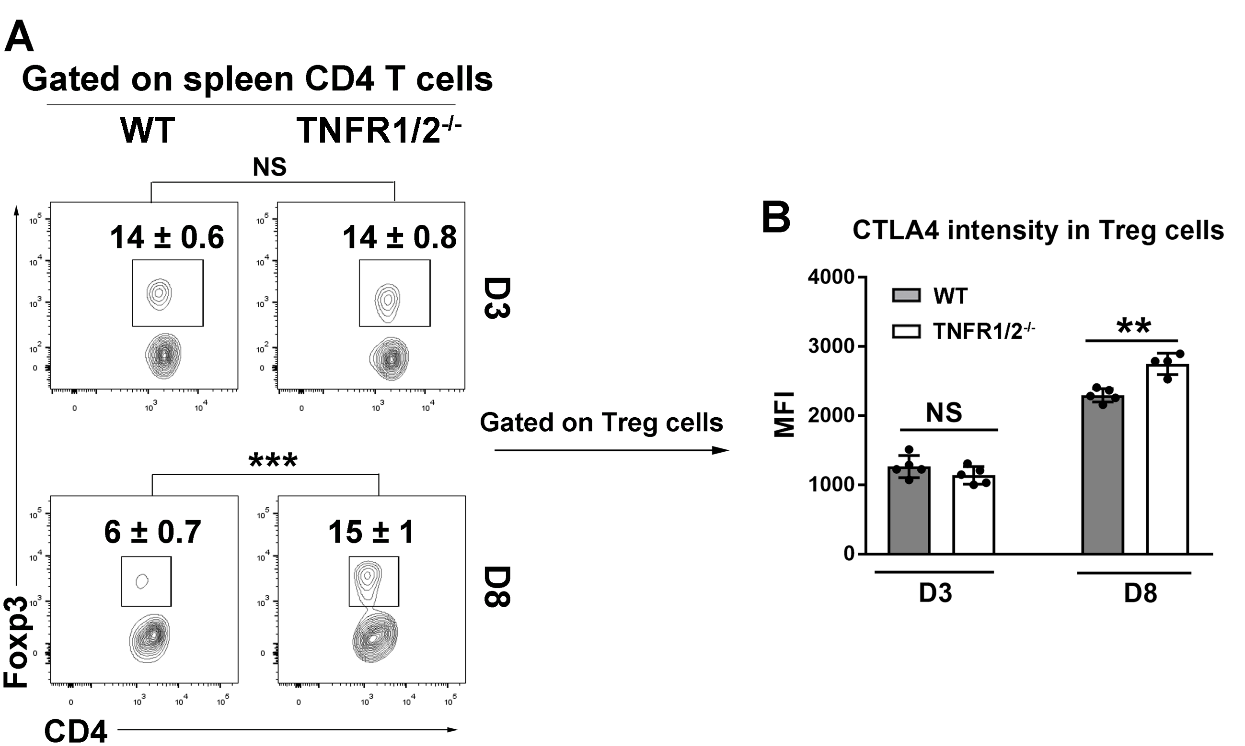
**

**Figure S4. Increased regulatory T (Treg) cells in the spleens of TNFR1/2-deficient mice.** WT and TNFR1/2-deficinet mice were infected *i.v.* with bacteria (6 × 10^4^ FFU) and sacrificed at D3 and D8. Splenocytes were stained and analyzed by flow cytometry. (A) Treg cells in the spleens. (B) CTLA4 expression on Treg cells. A two-tailed student t test was used. **, *p*<0.01; ***, *p*<0.001; NS, not significant.


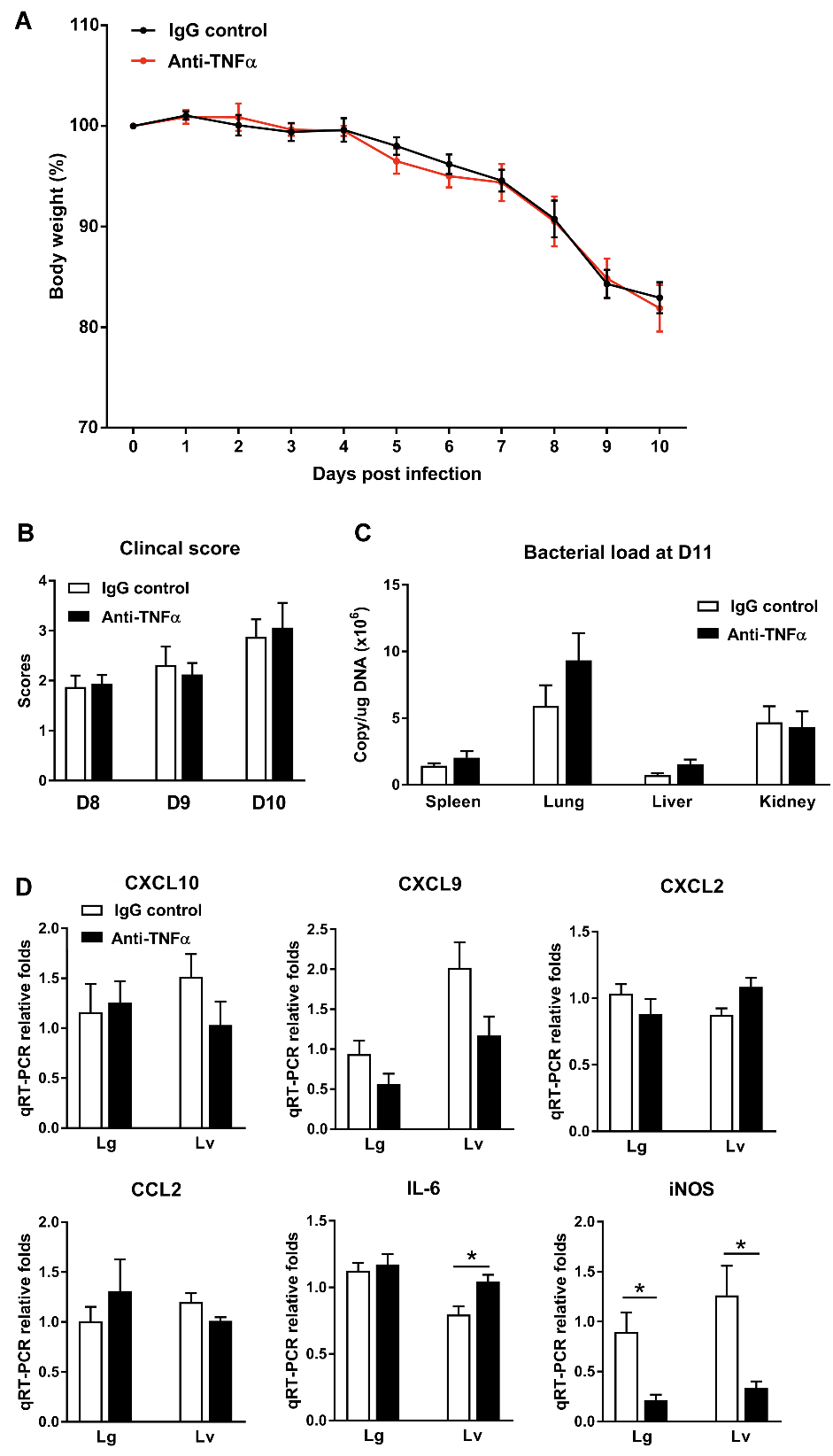


**Figure S5. Neutralization of TNF-α at the peak of infection was dispensable for disease severity.** B6 mice were infected *i.v.* with bacteria (6 × 10^4^ FFU) and intraperitoneally injected with anti-TNF-α antibody daily starting at D6 (200 μg/mouse). The percentages of body weight loss (A) and clinical scores (B) were recorded daily. (C) Mice were euthanized at D11 for measuring tissue bacterial loads by qPCR. (D) The mRNA levels of inflammatory genes in the lungs (Lg) and liver (Lv) were analyzed by qRT-PCR. A two-tailed student t test was used. *, *p*<0.05.
