# Supplementary material for: Distinct Role of TNFR1 and TNFR2 in Protective Immunity Against Orientia tsutsugamushi Infection in Mice
Source: Front Immunol. 2022 Apr 11;13:867924. doi: 10.3389/fimmu.2022.867924 (PMC9035742; doi:10.3389/fimmu.2022.867924)
Supplement: Supplementary file 2 [file Table_1.docx]

| **Supplementary table 1** | | | |
| --- | --- | --- | --- |
|  |  |  |  |

**Real-time PCR primers of murine genes**

Forward (5’ to 3’) Reverse (5’ to 3’)

IL-1β GCAACTGTTCCTGAACTCAACT ATCTTTTGGGGTCCGTCAACT

CXCL1 CTGGGATTCACCTCAAGAACATC CAGGGTCAAGGCAAGCCTC

CXCL2 CCAACCACCAGGCTACAGG GCGTCACACTCAAGCTCTG

CXCL9 GGAGTTCGAGGAACCCTAGTG GGGATTTGTAGTGGATCGTGC

CXCL10 CCAAGTGCTGCCGTCATTTTC GGCTCGCAGGGATGATTTCAA

IFN-γ ATGAACGCTACACACTGCATC CCATCCTTTTGCCAGTTCCTC

IL-6 TAGTCCTTCCTACCCCAATTTCC TTGGTCCTTAGCCACTCCTTC

IL-10 GCTCTTACTGACTGGCATGAG CGCAGCTCTAGGAGCATGTG

iNOS GTTCTCAGCCCAACAATACAAGA GTGGACGGGTCG ATGTCAC

CCL2 TTAAAAACCTGGATCGGAACCAA GCATTAGCTTCAGATTTACGGGT

GAPDH TGGAAAGCTGTGGCGTGAT TGCTTCACCACCTTCTTGAT
